# Differences in cardiovascular toxicities associated with cigarette smoking and snuff use revealed using novel zebrafish models

**DOI:** 10.1242/bio.018812

**Published:** 2016-06-22

**Authors:** Maggie Folkesson, Natalia Sadowska, Svante Vikingsson, Matts Karlsson, Carl-Johan Carlhäll, Toste Länne, Dick Wågsäter, Lasse Jensen

**Affiliations:** 1 Division of Drug Research, Department of Medical and Health Sciences, Linköping University, 581 85 Linköping, Sweden; 2 Division of Cardiovascular Medicine, Department of Medical and Health Sciences, Linköping University, 581 85 Linköping, Sweden; 3 Division of Applied Thermodynamics and Fluid Mechanics, Department of Management and Engineering, Linköping University, 581 83 Linköping, Sweden

**Keywords:** Zebrafish, Aneurysm, Aorta, Tobacco, Snuff, Cardiovascular

## Abstract

Tobacco use is strongly associated with cardiovascular disease and the only avoidable risk factor associated with development of aortic aneurysm. While smoking is the most common form of tobacco use, snuff and other oral tobacco products are gaining popularity, but research on potentially toxic effects of oral tobacco use has not kept pace with the increase in its use. Here, we demonstrate that cigarette smoke and snuff extracts are highly toxic to developing zebrafish embryos. Exposure to such extracts led to a palette of toxic effects including early embryonic mortality, developmental delay, cerebral hemorrhages, defects in lymphatics development and ventricular function, and aneurysm development. Both cigarette smoke and snuff were more toxic than pure nicotine, indicating that other compounds in these products are also associated with toxicity. While some toxicities were found following exposure to both types of tobacco product, other toxicities, including developmental delay and aneurysm development, were specifically observed in the snuff extract group, whereas cerebral hemorrhages were only found in the group exposed to cigarette smoke extract. These findings deepen our understanding of the pathogenic effects of cigarette smoking and snuff use on the cardiovascular system and illustrate the benefits of using zebrafish to study mechanisms involved in aneurysm development.

## Abstract (precis)

**Summary:** We analyze common and distinct developmental and cardiovascular toxicities caused by cigarette smoke and snuff, and include a new zebrafish embryo model for studying abdominal aortic aneurysm.

## INTRODUCTION

The World Health Organization (WHO) has recently reported that tobacco use is responsible for premature mortality in more than six million people annually. Cardiovascular diseases are the most common cause of mortality in the world and tobacco use is the principal contributor to the development of cardiovascular diseases including myocardial infarction, stroke, heart failure and aortic aneurysms (Ezzati et al., 2005; Kontis et al., 2015).

There are about 2500 substances in tobacco and this figure rises to 4000 different chemicals upon combustion (Adam et al., 2005). The content of smoke is directly introduced into the alveoli of the lung during smoking, and within seconds crosses into the blood stream and reaches the brain. Smoking delivers the substances more effectively than intravenous administration (Hukkanen et al., 2005). This may be one reason why nicotine replacement products are not very popular among smokers (Dome et al., 2010).

While smoking is the most common use of tobacco, keeping tobacco in the oral cavity for prolonged periods of time (chewing or snuffing) is also popular, especially in some societies. Both types of tobacco use lead to numerous compounds with potentially harmful effects, including nicotine, being taken up by the organism.

One of the toxic effects of chemicals present in tobacco products including cigarettes and snuff is degradation of elastin (Eurlings et al., 2014). Elastin is a protein that facilitates elasticity of various organs such as skin, arteries and heart valves. In humans all elastin is produced during embryogenesis with no regeneration after this period. Elastin in healthy individuals has a half-life of 40 years (Rucker et al., 1977), but due to the upregulation of elastin-degrading proteases and alteration of elastin fibers in tobacco users, the half-life is much shorter in this group of people (Just et al., 2007). Abdominal aortic aneurysm (AAA) is a common cardiovascular disease coupled to loss of elastin, which is strongly correlated to smoking (Landenhed et al., 2015).

Snuff use is a common practice in Scandinavia, with a presently poorly understood toxicity profile and unknown effect on the risk of AAA development. Snuff use is not associated with pulmonary pathologies, one of the most obvious toxicities associated with smoking. Compared to smoking cigarettes, snuff use does not lead to as high a risk of myocardial infarction (Huhtasaari et al., 1992, 1999; Hergens et al., 2005). However, the use of snuff raises the hazard ratio for coronary heart disease (Johansson et al., 2005). This could be due to the facts that snuff contains different chemicals to cigarettes, that the tobacco packages are kept longer in the person compared to cigarette smoke such that chemicals with other absorption profiles may take effect, or that the administration route of the snuff-derived chemicals is through the digestive tract rather than through the respiratory tract. The use of snuff, as well as other non-smoked tobacco products, by millions of people world-wide, coupled to the known risks associated with chemicals found in tobacco for the development of cardiovascular diseases, makes it imperative to develop a better understanding of how snuffing affects the cardiovascular system.

Zebrafish are particularly well-suited for studies of the toxicological effects of compounds such as those found in cigarette smoke (Ellis et al., 2014). They have also been frequently used to investigate toxicity of complex mixtures of chemicals in organic extracts (Bluhm et al., 2014), and for cardiovascular pathologies associated with tobacco use (Palpant et al., 2015), other toxic substances (Bayat et al., 2015) or genetic factors (Berndt et al., 2014). Due to the transparency and extra-uterine development of the zebrafish embryo, pathophysiological changes in, for example, heart rate, ventricular stroke volume and cardiac output can be readily investigated from videos of living embryos taken under the light microscope (Berndt et al., 2014). Such effects can often be complicated to study in other systems. Also, due to the facilitated, passive uptake of water-borne chemicals through the gills or skin (Ochoa-Alvarez et al., 2015), coupled to intra-vital imaging of the heart and aorta (Banjo et al., 2013), researchers may follow the development of the cardiovascular toxicities over time in embryos exposed to potentially toxic chemicals. These strengths of the system provide a strong argument for the more widespread use of zebrafish as an *in vivo* tool to study cardiovascular pathologies associated with toxic chemicals such as those present in tobacco products.

Here we use zebrafish embryos to provide a comprehensive view into the toxic effects of smoke and snuff extracts during vertebrate development. We have focused on elucidating differences in the toxicity pattern caused by cigarette smoke and snuff with a particular attention given to the development of cardiovascular pathologies. Furthermore, we have developed a zebrafish model for studying AAA, which have been lacking in the past, allowing us to examine the role of cigarette smoke and snuff during the early stages of AAA development. As such, we present here for the first time a comprehensive view of specific and overlapping cardiovascular toxicities associated with cigarette smoking or snuff use.

## RESULTS

### Tobacco smoke and snuff extracts exhibit concentration-dependent early embryonic mortality in zebrafish

**Fig. 1.**
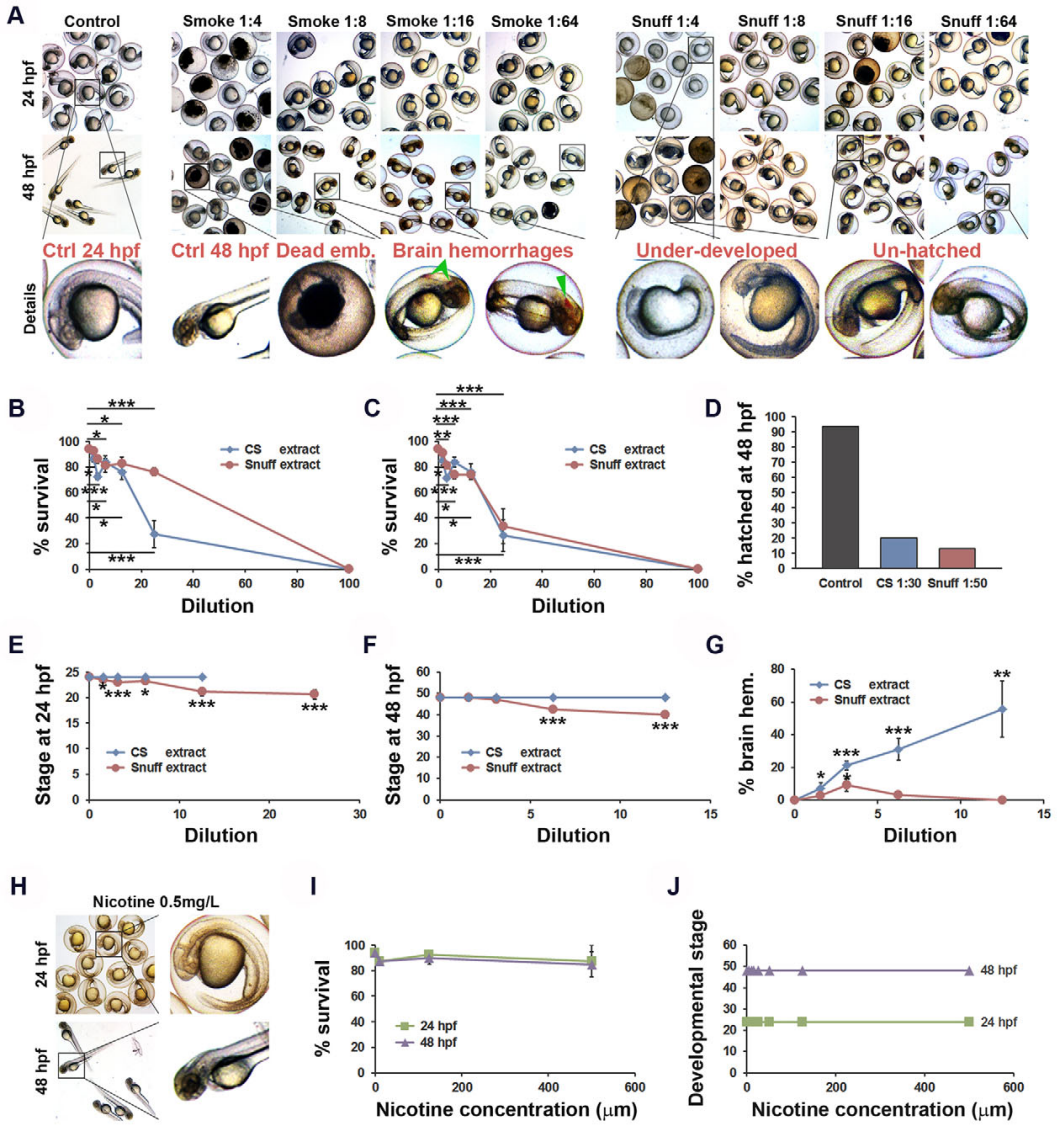
**Developmental toxicities of cigarette smoke and snuff extracts.** (A) Bright-field micrographs of zebrafish embryos at 24 or 48 hours post-fertilization (hfp) treated with nothing (control) or various concentrations of cigarette smoke (CS) or snuff extracts. Areas indicated by black boxes in the upper two rows are enlarged in the third row. Brain hemorrhages are highlighted by green arrowheads. (B) Quantification of the proportion of CS or snuff extract-treated embryos that survived to the 24 hpf stage. *n*=40 for the undiluted CS and snuff groups, 120 for control groups and 80 embryos for all other groups. CS: *P*<5.1×10^−11^, snuff: *P*<9.6×10^−10^ by single-factor ANOVA. *P<0.05, ***P<0.001 comparing each concentration of tobacco extract to its own control. (C) Quantification of the proportion of CS or snuff extract-treated embryos that survived to the 48 hpf stage. *n*=40 for the undiluted CS and Snuff groups, 120 for control groups and 80 embryos for all other groups. CS: *P*<7.7×10^−9^, snuff: *P*<4.1×10^−9^ by single-factor ANOVA. *P<0.05, ***P<0.001 comparing each concentration of tobacco extract to its own control. (D) Quantification of the proportion of non-treated, CS extract-treated or snuff extract-treated embryos hatched at the 72 hpf stage. *n*=15 embryos. (E) Average stage translated to time-after-fertilization of embryos treated with CS or snuff extracts evaluated 24 hpf. *n*=36 embryos for control (0), 38, 36, 34, 30 and 29, or 35, 30, 36 and 34 embryos for increasing concentrations of snuff or CS, respectively. CS: *P*>0.05, snuff: *P*<7.5×10^−7^ by single-factor ANOVA. *P<0.05, ***P<0.001. (F) Average stage translated to time-after-fertilization of embryos treated with CS or snuff extracts evaluated 48 hpf. *n*=36 embryos for control (0), 37, 32, 29 and 28, or 35, 30, 36 and 34 embryos for increasing concentrations of snuff or CS, respectively. CS: *P*>0.05, snuff: *P*<3.2×10^−25^ by single-factor ANOVA. ***P<0.001. (G) Quantification of the proportion of CS extract-treated or snuff extract-treated embryos exhibiting obvious bleedings in the brain (brain hem.) at 48 hpf. *n*=80 embryos. SC: *P*<0.018, snuff: *P*<6.3×10^−4^ by single-factor ANOVA. *P<0.05, **P<0.01, ***P<0.001. (H) Bright-field micrographs of zebrafish embryos at 24 or 48 hpf treated with 0.5 mg/l nicotine. Areas indicated by black boxes in left column are enlarged in the right column. (I) Quantification of the proportion of nicotine-treated embryos that survived to the 24 or 48 hpf stages. *n*=120 embryos for control (0) and 80, 40 or 40 for increasing concentrations of nicotine. *P*<0.015 by single-factor ANOVA. (J) Average stage translated to time-after-fertilization of embryos treated with nicotine evaluated 24 or 48 hpf. *n*=36 embryos for control (0) and 35, 34, 34, 32, 32, 37 and 35, or 35, 34, 31, 32, 31, 36 and 34 embryos at 24 hpf or 48 hpf, respectively. *P*>0.05 by single-factor ANOVA. All experiments were repeated twice with similar results. Results are shown as means±s.e.m. and statistical evaluation as indicated by asterisks was done using Student’s two-tailed *t*-test assuming equal variance between the groups.

To investigate the gross toxicities associated with exposure to cigarette smoke or snuff, zebrafish embryos were subjected to either undiluted or diluted cigarette smoke or snuff extracts (Fig. S1) as indicated and examined for the development of toxic phenotypes at 24 and 48 hours post-fertilization (hpf). Undiluted extracts exhibited immediate and complete mortality and was therefore not evaluated further (Fig. 1A-C). A 1:4 dilution of cigarette smoke extract (CS) also lead to mortality in 73% of the embryos already after 24 h exposure, whereas lower concentrations were generally compatible with embryo survival at both 24 and 48 h of exposure (Fig. 1A-C). Undiluted snuff extract was also lethal and 1:4 dilutions led to mortality of 66% of the embryos after 48 h of exposure. We noticed, however, that the mortality of embryos exposed to 1:4 dilution of snuff extract was relatively low after the first 24 h (Fig. 1A-C). The LD50 at 24 h was calculated to be 18% dilution for CS and 64% dilution for snuff, or at 48 h 18% dilution for CS and 19% dilution for snuff.

### Snuff extract causes developmental retardation of zebrafish embryos

Using HPLC we determined that undiluted snuff extract contained 500 µM of nicotine, indicating that we retrieved approximately 1300 µg nicotine per snuff bag. The total nicotine content per bag was according to the manufacturer 9600 µg, indicating that the extraction efficiency of nicotine and probably other chemicals with similar hydrophilicity was 13.5%. Undiluted cigarette smoke extract contained 18.7 µM nicotine, indicating that we retrieved approximately 75 µg nicotine per cigarette. The manufacturer discloses that the total nicotine content per cigarette was 1000 µg, leading to an extraction efficiency of 7.5%. Due to these differences in extraction efficiency, and toxicities associated with high concentrations of the extracts, we decided to continue our investigations using a dilution of 1:30 cigarette smoke extract (corresponding to 0.25% of the total amount of nicotine-like chemicals from the product) and 1:50 snuff extract (corresponding to 0.27% of the nicotine-like chemicals from the product).

Interestingly, while the embryos survived the snuff extract better than the cigarette smoke extract, it seemed that their development was slowed down by up to 48 h. While both cigarette smoke and snuff extract impaired hatching at 72 hpf (Fig. 1D), cigarette smoke extract-treated embryos seemed to otherwise have developed at the same rate as control embryos, regardless of the concentration used (Fig. 1A,E,F). Carefully staging the embryos according to Kimmel et al. (1995), however, we found that at concentrations between 3% and 25% of the stock (1:32-1:4 dilution), snuff extract-treated embryos exhibited significantly retarded development (Fig. 1A,E,F). In this group development proceeded at a slower pace, i.e. snuff-treated embryos were at the 19-hpf stage 24 h after egg fertilization, and at the 38 hpf stage 48 h after egg fertilization. This developmental retardation was not associated with additional toxicities compared to CS-treated embryos, which developed at the expected pace (Fig. 1A,E,F). These findings indicate that chemicals specifically present in snuff but not in cigarettes are associated with developmental retardation in zebrafish.

### Cigarette smoke but not snuff, causes cerebral hemorrhaging during development

In contrast to snuff-treated embryos, CS treatment was associated with frequent development of large cerebral hemorrhages at 48 hpf, in a concentration-dependent manner (Fig. 1G). The IC50 of hemorrhagic strokes was calculated as 11% dilution of the CS extract stock. An analysis of the snuff extract-treated embryos clearly showed that this phenomenon did not apply to this group regardless of the concentration used. This effect was also not seen in embryos treated with pure nicotine (Fig. 1H), and therefore seems to be specifically associated with chemicals present in smoked tobacco products. Nicotine treatment at concentrations up to 500 µM also did not affect embryo survival or the rate of development (Fig. 1I,J) indicating that nicotine is not the main cause of embryonic lethality, retardation or hemorrhagic stroke formation in zebrafish.

### Tobacco extracts cause defects in zebrafish lymphatic development

**Fig. 2.**
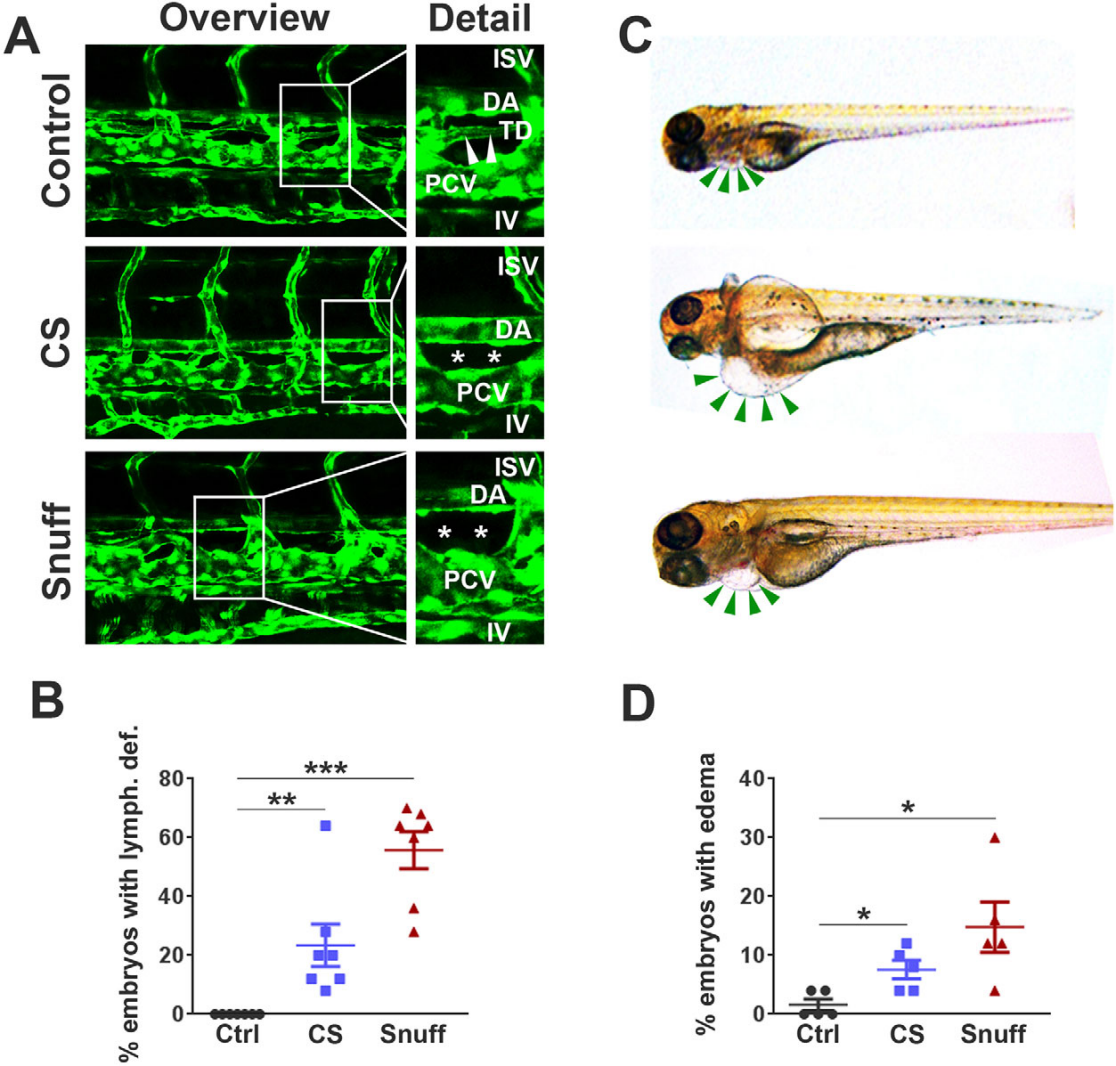
**Defective development of lymphatic vessels in zebrafish embryos treated with cigarette smoke extract or snuff extract.** (A) Confocal micrographs of the central trunk region in *fli1a:EGFP* transgenic zebrafish at 4 days post-fertilization (dpf) treated with nothing (control), cigarette smoke extract (CS) at 1:30 dilution or snuff extract at 1:50 dilution. White boxes in the left column represent the areas enlarged in the right column. ISV, inter-segmental vessel; DA, dorsal aorta; TD, thoracic duct; PCV, posterior cardinal vein; IV, intestinal vein. White stars represent the area in which the thoracic duct should have appeared but is lacking. (B) Quantification of the proportion of non-treated, CS extract-treated or snuff extract-treated embryos with an absent or major defect in TD development at 4 dpf. *n*=160 embryos. **P<0.01, ***P<0.001. (C) Bright-field micrographs of zebrafish larvae at 4 dpf treated with nothing (top image), CS extract (middle image) or snuff extract (bottom image). Green arrows indicate the pericardium, with pericardial edema visible in the CS and snuff groups. (D) Quantification of the proportion of non-treated, CS extract-treated or snuff extract-treated embryos with pericardial edema at 4 dpf. *n*=110 embryos. *P<0.05. All experiments were repeated twice with similar results. Results are shown as means±s.e.m. and statistical evaluation was done using Student’s two-tailed *t*-test assuming equal variance between the groups.

We thereafter investigated the effects of CS or snuff extract on the developing vasculature. We did not observe any obvious changes in angiogenesis at 4 days post-fertilization (dpf) as evidenced by the normal morphology of intersegmental, intestinal and other peripheral vessels (Fig. 2A). We did, however, observe a distinct lack of the thoracic duct, the main lymphatic vessel which in control embryos were clearly developed and present between the dorsal aorta (DA) and posterior cardinal vein (PCV) at 4 dpf in both groups (CS and snuff). The lack of properly developed lymphatic vessels was most prevalent in the snuff extract-treated embryos (Fig. 2A,B). Such defects in lymphatic development were, as expected, associated with pronounced pericardial and in some cases yolk sac edema (Fig. 2C,D). These findings suggest that tobacco products have detrimental effects on developmental lymphangiogenesis but not angiogenesis in zebrafish embryos.

### Tobacco extracts cause impaired cardiac function

**Fig. 3.**
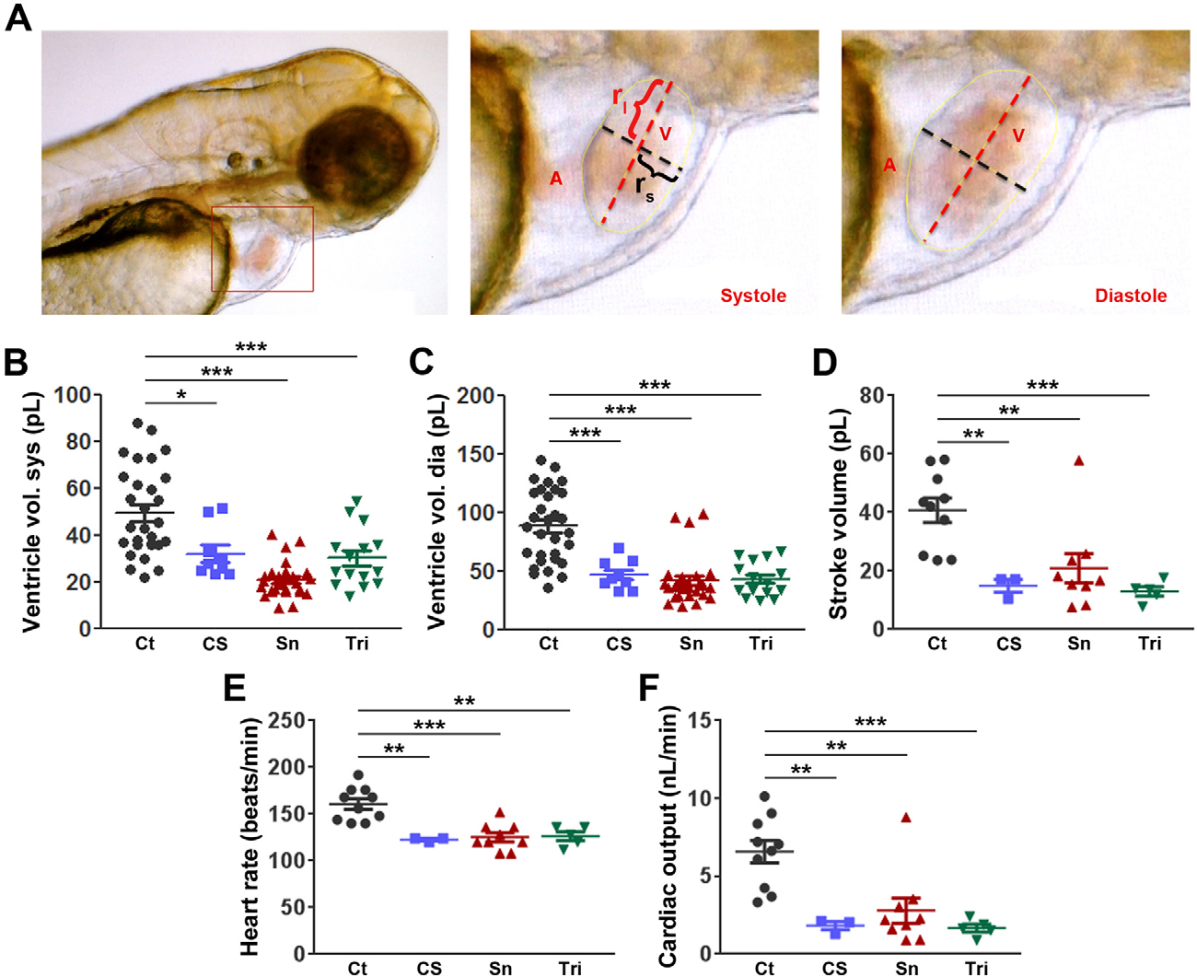
**Lowered heart rate, ventricular dimensions, stroke volume and cardiac output in zebrafish embryos treated with cigarette smoke or snuff extracts.** (A) Still images from a microscope video of a zebrafish larvae at 3 days post-fertilization (dpf). The area indicated by a red box in the image to the left is enlarged and shown at end systole or end diastole in the middle or right images, respectively. A, Atrium; r_l_, long-axis radius; r_s_, short-axis radius; V, volume. (B,C) Quantification of the average volume of the ventricle at systole (B) or diastole (C) in 3 dpf embryos treated with nothing, cigarette smoke extract at 1:30 dilution (CS), snuff extract at 1:50 dilution (Sn) or the anesthetic tricaine (Tri) at 0.2 mg/ml. *n*=10, 3, 9 and 5 embryos (three measurements per embryo) in the control, CS, SN and Tri groups respectively. *P<0.05, ***P<0.001. (D) Quantification of the stroke volume in picoliters (pl) of the same 3 dpf embryos as in B and C. **P<0.01, ***P<0.001. (E) Quantification of the average heart rate in beats per minute (beats/min) of the same 3 dpf embryos as in B and C. **P<0.01, ***P<0.001. (F) Quantification of the cardiac output in nanoliters per minute (nl/min) of the same 3 dpf embryos as in B and C. **P<0.01, ***P<0.001. The experiment was repeated with similar results. Results are shown as means±s.e.m. and statistical evaluation was done using Student’s two-tailed *t*-test assuming equal variance between the groups.

While chronic tobacco use is known to affect heart function in adults (Linneberg et al., 2015) less is known about the effects of cigarette smoke or snuff on the development of ventricular performance. We investigated this using living, immobilized zebrafish embryos at 3 dpf, treated with either cigarette smoke or snuff extracts compared to non-treated controls. In general, we observed that mounting the zebrafish embryos in low-melting agarose led to a slight increase in ventilation frequency indicating a hypoxic response in such embryos compared to freely swimming embryos (results not shown). However, the heart rate, stroke volume and cardiac output of such mounted embryos at 3 dpf were similar to those described in other studies (Fig. 3E) (Yozzo et al., 2013). Exposure to CS or snuff for only 24 h (from the 48 hpf to the 72 hpf stage) led to a reduction in ventricular dimensions as indicated by lower ventricular volumes at both systole and diastole (Fig. 3A-C), and a dramatic decrease in heart rate, stroke volume and cardiac output (Fig. 3D-F), similar to that seen when using the anesthetic tricaine at 0.2 mg/ml, a drug known to induce cardiac arrest at high concentrations in zebrafish embryos (Nicoli et al., 2010). These findings demonstrate that tobacco extracts strongly inhibit development of normal ventricular functions.

### Prolonged exposure to snuff but not cigarette smoke extract leads to aneurysm formation

**Fig. 4.**
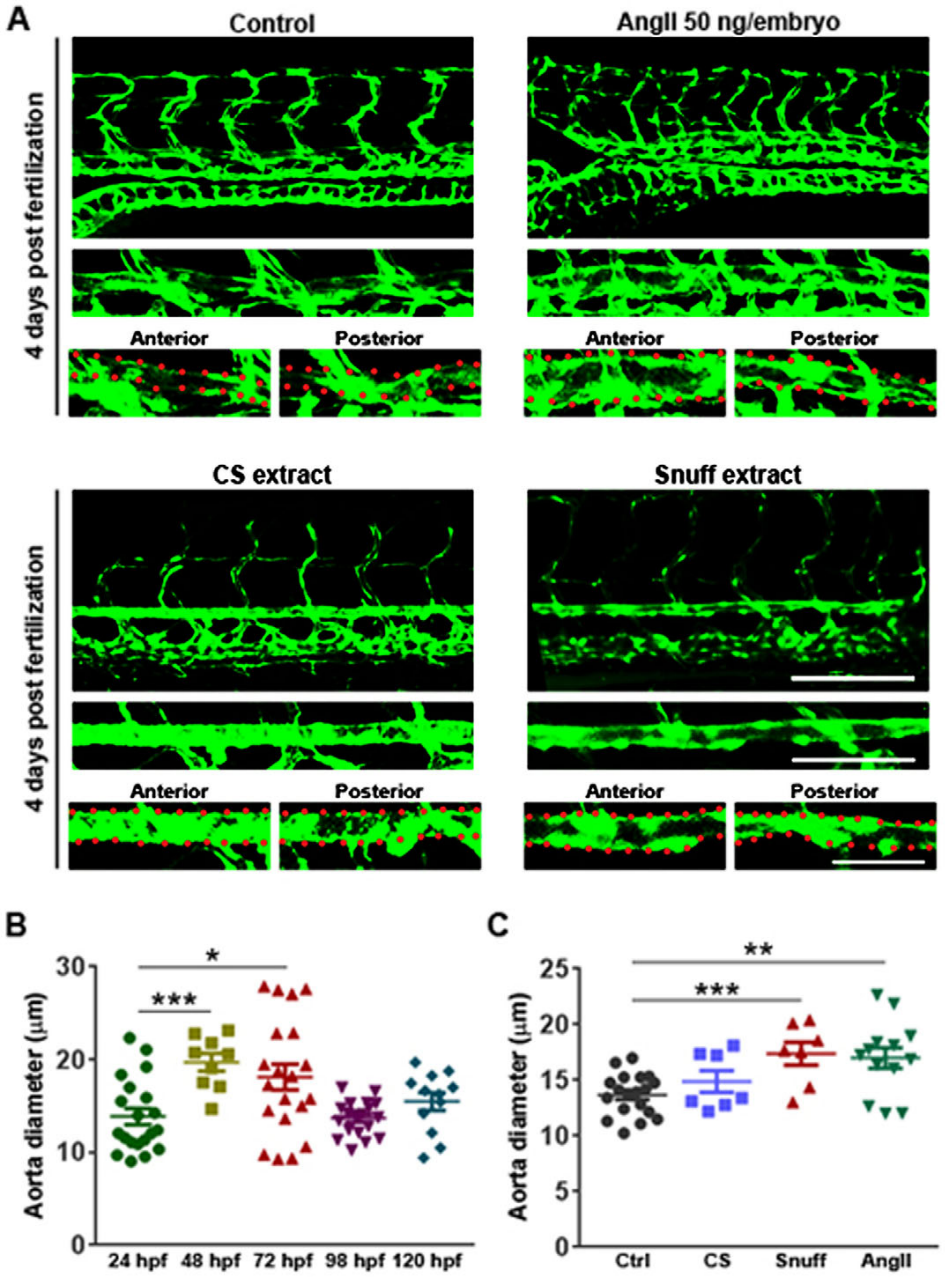
**Abdominal aortic aneurysm formation in zebrafish embryos following treatment with Angiotensin II or snuff extract.** (A) Confocal micrographs of the central trunk region in *fli1a:EGFP* embryos at 4 days post-fertilization (dpf) either non-treated (control), injected at the 1-cell stage with 50 ng angiotensin II (AngII) in the yolk, or treated with cigarette smoke extract (CS) at 1:30 dilution or snuff extract at 1:50 dilution from the 2 dpf stage. The aorta is highlighted in a magnified image under the overview image and the abdominal (anterior) and distal (posterior) parts of the aorta further magnified in the left and right images, respectively, under the isolated aorta image. Red dots outline the aortic wall. Scale bars: 100 µm, 50 µm and 50 µm in the overview, aorta highlight and posterior aorta highlight images, respectively. (B) Quantification of the average aorta diameter in the abdominal region (anterior aorta images shown in A) of control zebrafish embryos during development. *n*=20, 9, 20, 19 and 12 embryos at 24, 48, 72, 98 and 120 hpf, respectively. *P<0.05, ***P<0.001. (C) Quantification of the average aorta diameter in the abdominal region (anterior aorta images shown in A) of zebrafish embryos treated with CS, snuff or AngII as in A. *n*=19, 7, 7 and 13 in the control, CS, Sn and AngII groups respectively. **P<0.01, ***P<0.001. All experiments were repeated twice with similar results. Results are shown as means±s.e.m. and statistical evaluation was done using Student’s two-tailed *t*-test assuming equal variance between the groups.

In order to investigate if tobacco extracts could be involved in development of a second major class of cardiovascular diseases, namely aortic aneurysms, we first investigated the development of the aorta in terms of changes in its diameter from 24 hpf to 5 dpf. We found that during normal development of zebrafish embryos the diameter of the aorta increase significantly at day 2 and 3, compared to its size at day 1, perhaps as a result of increased cardiac output resulting from an increase in cardiac load due to the increased peripheral vascular resistance that emerges when smaller vessels, such as inter-segmental vessels (ISVs) and small cranial and brain vessels, are being perfused and included in the circulation at approximately 28-32 h (Fig. 4A,B). At 4 dpf, however, the aorta narrows, probably due to remodeling, with improved structure of the aortic wall including coverage with smooth muscle cells (Fig. 4A,B). At 5 dpf the diameter again increases, probably illustrating a new wave of increased cardiac load and the need for increased blood absorption and flow through the aorta. In healthy embryos, the diameter of the aorta from the start to the tip of the trunk is reasonably homogenous. (Fig. 4A). We found, however, that injecting embryos with 50 ng of AngII at the 1-cell stage induced a significant dilation of the aorta at 4 dpf, specifically in the site proximal and posterior to the swim bladder (Fig. 4A,C). This phenotype is strikingly similar to what is observed in arteriosclerosis-prone mice treated with AngII for 4-8 weeks, in which such aneurysms are commonly found in the abdominal region and therefore characterized as abdominal aortic aneurysms (AAA) (Daugherty et al., 2000). Interestingly, this region is likely associated with increased wall shear stress as it juxtaposes an approximately 45° bend of the aorta associated with the inflation of the swim bladder (Fig. 4A). Additionally, in rodents, AAAs specifically develop in a region associated with increased wall shear stress (Daugherty et al., 2000). These findings indicate that at least this critical aspect of the disease is accurately phenocopied by a single injection of AngII in zebrafish embryos.

Unexpectedly, treatment with CS from 2 to 4 dpf did not lead to a significant dilation of the aorta. Snuff treatment, however, led to a strong dilation of the aorta in the abdominal region, similar to the one found in AngII-injected embryos. These results introduce for the first time two novel methods to induce aneurysm in an organism to study pathogenesis of AAA and suggest that snuff treatment may increase the risk of AAA development.

## DISCUSSION

Growing scientific evidence from large epidemiological studies relate tobacco use to cardiovascular diseases (Ezzati et al., 2005; Kontis et al., 2015). Relatively few pre-clinical studies, however, have been conducted to thoroughly investigate specific developmental and cardiovascular toxicities induced by exposure to complex mixtures of chemicals found in tobacco products. Furthermore, snuff and other oral tobacco products contain considerably more nicotine, and an entirely different array of potentially harmful chemicals compared to smoked tobacco products, and therefore may affect disease risk differently than cigarette smoking. Currently, however, snuff and other non-smoked tobacco products are considered safe to use by many in the general public, a notion that is likely to be erroneous but difficult to contest due to lack of knowledge related to differences in toxicities between tobacco products, especially toxicities of oral products such as snuff. Here, for the first time, we have conducted a thorough comparison of developmental and cardiovascular toxicities observed in developing zebrafish embryos after exposure to various concentrations of extracts from cigarette smoke and snuff. We found that while very high, supraphysiological concentrations, as expected, lead to 100% early embryonic mortality, lower concentrations of snuff or CS caused specific developmental defects including developmental retardation and cerebral hemorrhage, respectively. Previous reports have investigated the toxicities caused by various types of cigarettes (Massarsky et al., 2015; Palpant et al., 2015; Ellis et al., 2014; Hammer et al., 2011), but did not identify cerebral hemorrhage as a major developmental defect, focusing rather on gross developmental malformations of the body plan (i.e. teratogenesis), and behavioral and cardiac toxicities caused by cigarette-derived chemicals. The highly reproducible and very large proportion of embryos that developed strokes in response to CS treatment is therefore an alarming new finding that deserves further study.

The toxic but sub-lethal concentrations of tobacco extracts investigated in this study are likely to mirror the levels of chemicals from such products in the blood of heavy smokers or snuff users. For example, cerebral hemorrhage, lymphedema and heart dysfunction was observed in embryos treated with a 1:30 dilution of a stock created by extraction of chemicals from 40 cigarettes in 1 liter of E3 medium. If such chemicals are absorbed to the same extent into the blood stream in the lungs of smokers as in our experimental setup, and if we consider an individual having a total blood volume of 5 liters, these concentrations would be reached following smoking of seven cigarettes, which is well below the daily intake of many users. A similar calculation for snuff would indicate that developmental retardation or AAA could be expected from the consumption of 19 or six bags of snuff, respectively. More research is needed on the metabolism and potential build-up of the harmful chemicals in the body over time in individuals smoking or using snuff daily, but these findings give new insights into potential developmental toxicities even by rather modest use of these tobacco products during pregnancy. Unexpectedly, these effects were not seen in embryos treated with pure nicotine, implying that other chemicals present in either snuff or cigarettes are responsible for the above-mentioned phenotypes observed in this study. These findings are corroborated in a recent report in which exposure to particulate matter from tobacco caused toxicities that similarly were not phenocopied by exposure to nicotine (Massarsky et al., 2015). Recently a thorough chemo-biology approach was taken to evaluate the role of various chemicals found in cigarettes on fetal development in smoking, pregnant women (Feltes et al., 2013). The phenotypes observed in this study, including both developmental retardation and neurotoxic effects, are also found in babies born to smoking mothers and associated with toxic interactions between at least 50 different substances from cigarettes found in high levels in the mothers blood (Feltes et al., 2013). While no such study has been done in mothers using snuff, this implies that our findings in zebrafish are translatable to human development.

Snuff, cigarette smoke and nicotine all led to defects in lymphatic development and lymphedema, which could mean that such toxicities could be due to nicotine alone. Using nicotine-free tobacco products could potentially verify this hypothesis. Both snuff and CS also impaired ventricular function as represented by reduced heart rate and lowered ventricular stroke volume, which therefore also could be due to nicotine found in these products. Such a hypothesis is strengthened by the observation that nicotine is an important regulator of ventricular development and function also in humans (Yan and D'Ruiz, 2015). However, CS reduced ventricular function more dramatically than snuff, whereas snuff contained more than tenfold higher nicotine levels, again suggesting that other chemicals present in both these tobacco products may be involved in precipitation of the observed ventricular deficiencies.

To study the role of CS or snuff on AAA *in vivo*, we have developed two new methods for inducing and studying AAA in zebrafish embryos through a single injection of AngII and exposure to snuff. We found that AAA developed at the 4 dpf stage, exhibiting the anatomical characteristics of AAA in the AngII mouse model of AAA and humans (Daugherty et al., 2000). Our zebrafish model of AAA showed aneurysm in a distinct abdominal region proximal and posterior to the swim bladder. Tobacco smoking in particular is the main environmental risk factor for abdominal aortic aneurysm (Norman and Curci, 2013) but thus far no animal models of aneurysm have been generated using only tobacco as a driver. We did not find AAA in zebrafish embryos exposed to CS. This discrepancy compared to the situation in humans could be due to the relatively short exposure time to toxic chemicals found in the cigarettes in our model (48 h compared to years of exposure in humans) or that such CS chemicals act primarily on the developed/aged aorta and do not induce AAA during development. Indeed, AAA rarely appears in people under 48 year of age (Singh et al., 2001). Further work should evaluate the role of tobacco products including CS and snuff on AAA formation in adult/aged zebrafish. It also could be that aneurysms found in smokers may be attributed to other co-morbidities such as increased abdominal and vascular adipose tissue. Interestingly, we found that snuff induced aneurysms to the same extent as AngII injection, suggesting that snuff may be an important risk factor for aneurysm formation also in humans. This important finding, however, requires validation in human studies. These findings not only suggest that snuff use may be associated with AAA development, but also generally establishes zebrafish embryos at 4 dpf as a convenient, fast and attractive model system for studying mechanisms involved in aneurysm formation *in vivo*.

In conclusion we have here, using toxicity assays in zebrafish embryos, clearly shown that cigarettes and snuff lead to different developmental and cardiovascular toxicities, although with some overlap, which in most cases could not be attributed to the effects of nicotine alone. This is the first report clearly showing that snuff use is in some aspects more toxic than cigarettes, and the first report of specific toxicities associated with either type of tobacco product. In humans, further differences in organ-specific toxicities in particular are expected due to the difference in the administration route of the products. As the vast majority of the studies in this area have focused on toxicities associated with cigarette smoking and not snuff or other types of tobacco use, this may suggest to some individuals that snuff use is safe, as such specific, administration route-related toxicities would not apply, without taking other specific toxicities associated with snuff use into account. As such, it is extremely important to raise public awareness about the potential risks using snuff, which is particularly important considering the increase in laws related to banning of smoking (but not other types of tobacco use), associated with an increase in the use of alternative tobacco products including snuff all over the world. We hope our current study may help in this regard. Moreover, we here present a new model to study AAA. Through two novel methods we succeeded in inducing abdominal aortic aneurysm in zebrafish embryos which potentially could give researchers a powerful and feasible tool for understanding mechanisms related to this lethal and common disorder among smokers.

## MATERIAL AND METHODS

### Animals

Adult (3-18 months) male and female *Fli1a:EGFP* transgenic zebrafish (*Danio rerio*) (Lawson and Weinstein, 2002) were maintained at 28.5°C and subjected to 14-h light/10-h dark cycle in our zebrafish facility at Linköping University. Embryos were produced by natural mating and kept in a humidified incubator at 28.5°C. All studies were performed in accordance with guidelines approved by the Linköping research animal ethical council.

### Production of tobacco extracts

Snuff extracts were produced by placing 60 snuff packs (Göteborgs Rapé) in 1000 ml E3 medium for 1 h at 37°C under rigorous stirring. The resulting crude extract was filtered through a 0.2 µm filter in order to remove undissolved particulate matter and kept at 4°C until use. Cigarette smoke extract was produced by coupling 40 lit cigarettes (Marlboro regular) serially to a vacuum system in which the smoke was guided through 1000 ml of E3 medium. The cigarette smoke extract was also filtered through a 0.2 µm filter and kept at 4°C until use. Pure nicotine (Sigma Aldrich) was used at 6.163 M and diluted to the indicated final concentration in E3 water.

### HPLC determination of nicotine content

Determination of the concentration of nicotine in tobacco extracts was done by HPLC as previously described (Ljungberg et al., 2013). Briefly, 5 ml samples of either snuff or cigarette smoke extract were diluted in 45 ml of water and the concentration of nicotine determined running 20 µl of the diluted sample on an X-bridge C18 3 μm, 3×100 mm column from Waters (Milford, MA, USA). Samples were separated using a mobile phase consisting of 5:95 (v/v) acetonitrile:ammonium formiate 10 mM, pH 4.2, at a flow rate of 500 μl/min. Nicotine, detected at 260 nm, was quantified by comparison to a standard curve using pure nicotine.

### Developmental toxicity assay

At 4 hours post-fertilization (hpf), 20 embryos were incubated in 60 mm diameter tissue culture dishes with 15 ml of E3 embryo medium containing 0.003% 1-phenyl-2-thiourea (PTU) and various concentrations of snuff extract, cigarette smoke extract or pure nicotine. The embryos were kept at 28.5°C for 24 to 72 h as indicated until analyzed under a stereomicroscope (Nikon SMZ1500). Images were obtained using NIS-Elements F software (Nikon). The hatching rate, mortality rate and various types of developmental defects were recorded. LD50 values were calculated from the best-fitting linear trendline in percentage tobacco extract and percentage dead embryo plots, solving for y=50. Kimmel's technique for staging embryonic development (Kimmel et al., 1995) was used to reveal effects on developmental retardation. Briefly, 24 hpf staging was done by counting the number of chevron-shaped somites, whereas at 48 hpf staging was done by various measures including somite number, head-body angle, tissue characteristics (including formation/remodeling of brain and caudal structures) and the size and shape of the yolk extension.

### Zebrafish lymphedema assay

Zebrafish embryos were exposed to cigarette smoke or snuff extracts from 48 hpf to 4 days post-fertilization (dpf), 10 embryos per group, anesthetized with 0.5 mg/ml ethyl 2-aminobenzoate (Sigma) and mounted in 1% low melting agarose on a microscope cover slip. The central part of the trunk between the swim bladder and the end of the GI tract were imaged using a LSM700 Zeiss Inverted Confocal microscope with associated software, taking stacks with 5 µm between each slide and 20-30 slides per embryo, covering the entire thickness of the trunk. The presence or absence of a continuous vessel running between the dorsal aorta and posterior cardinal vein (i.e. the thoracic duct) was scored by visual inspection of the resulting micrographs. Clear bulbous fluid-filled spaces around the heart (pericardial edema) or yolk (yolk sac edema) was scored by visual inspection of light micrographs using a Nikon SMZ1500 microscope and NIS-Elements F software.

### Zebrafish aneurysm assay

Zebrafish embryos were injected with 1 nl Angiotensin II (AngII, 50 mg/ml) at the 1-cell stage. Fertilized embryos were selected at 4 hpf and incubated at 28.5°C in PTU-containing E3 medium for 4 days, and then visualized by confocal microscopy. Uninjected embryos were either non-treated (control) or treated with cigarette smoke extract (1:50) or snuff extract (1:30) in PTU-containing E3 medium from the 48 hpf stage to the 4 dpf stage and then analyzed by confocal microscopy. The diameter of the aorta was measured in confocal *z*-stack projections acquired as described in the lymphedema assay above, using ImageJ software (NIH) along the entire length spanning the swim bladder to the anal opening, and the largest diameter used for further analysis.

### Determination of heart function

Heart rate, ventricular stroke volume and cardiac output were determined from videos taken under the light microscope (SMZ-1500, Nikon) essentially as described (Hoage et al., 2012). Briefly, heart rate (beats per minute) was calculated by counting the number of beats over a period of 20-30 s. To calculate the ventricular stroke volume, long-axis (lax) and short-axis (sax) ventricular diameters were measured from still images at end systole and end diastole using ImageJ, and used to calculate the ventricular volume by the formula V=0.5×sax^2^×lax. The still images were taken from videos of the beating heart in living embryos at the time when the ventricle was the most contracted (end systole) or most dilated (end diastole) using windows movie-maker, with three pairs of end systole or end diastole images being used per embryo. The stroke volume was taken as V_end diastol__ic_−V_end systol__ic_. The cardiac output was calculated as heart rate (in beats per minute)×stroke volume.

### Statistics

Results were generated in an un-blinded fashion and shown as means±s.e.m. from representative experiments that were repeated at least two times to confirm the findings. Statistical comparisons between two individual data sets were made using two-tailed, unpaired Student’s *t*-test assuming equal variance between the groups. Unless indicated otherwise, comparisons have been made against the control group (the left-most group in all the graphs) or between multiple data sets using single-factor ANOVA. Significance is indicated as *P*-values (ANOVA) or using the following symbols when comparing two individual data sets in the graphs (*t*-test): *P<0.05, **P<0.01 and ***P<0.001, with *n*-values as indicated in the figure legends representing the number of individual embryos being analyzed. Sample sizes were determined based on expected differences and variations between groups obtained in pilot experiments as well as using positive and negative controls. Embryos with severe developmental deformities were excluded from the analysis of heart function and lymphatic and AAA development. Healthy embryos were randomly allocated from a combined pool of embryos to each experimental group at the beginning of all experiments.
